# Mechanical Genomic Studies Reveal the Role of d-Alanine Metabolism in Pseudomonas aeruginosa Cell Stiffness

**DOI:** 10.1128/mBio.01340-18

**Published:** 2018-09-11

**Authors:** Rishi R. Trivedi, John A. Crooks, George K. Auer, Joel Pendry, Ilona P. Foik, Albert Siryaporn, Nicholas L. Abbott, Zemer Gitai, Douglas B. Weibel

**Affiliations:** a Department of Biochemistry, University of Wisconsin—Madison, Madison, Wisconsin, USA; b Department of Biomedical Engineering, University of Wisconsin—Madison, Madison, Wisconsin, USA; c Department of Chemical Engineering, University of Wisconsin—Madison, Madison, Wisconsin, USA; d Department of Physics & Astronomy, University of California, Irvine, Irvine, California, USA; e Department of Molecular Biology & Biochemistry, University of California, Irvine, Irvine, California, USA; f Department of Molecular Biology, Princeton University, Princeton, New Jersey, USA; g Department of Chemistry, University of Wisconsin—Madison, Madison, Wisconsin, USA; University of Texas at Austin; Georgia Institute of Technology School of Biological Sciences

**Keywords:** cell stiffness, DadA, mechanical genomics, *Pseudomonas aeruginosa*, cell wall

## Abstract

The stiffness of bacteria prevents cells from bursting due to the large osmotic pressure across the cell wall. Many successful antibiotic chemotherapies target elements that alter mechanical properties of bacteria, and yet a global view of the biochemistry underlying the regulation of bacterial cell stiffness is still emerging. This connection is particularly interesting in opportunistic human pathogens such as Pseudomonas aeruginosa that have a large (80%) proportion of genes of unknown function and low susceptibility to different families of antibiotics, including beta-lactams, aminoglycosides, and quinolones. We used a high-throughput technique to study a library of 5,790 loss-of-function mutants covering ~80% of the nonessential genes and correlated P. aeruginosa individual genes with cell stiffness. We identified 42 genes coding for proteins with diverse functions that, when deleted individually, decreased cell stiffness by >20%. This approach enabled us to construct a “mechanical genome” for P. aeruginosa. d-Alanine dehydrogenase (DadA) is an enzyme that converts d-Ala to pyruvate that was included among the hits; when DadA was deleted, cell stiffness decreased by 18% (using multiple assays to measure mechanics). An increase in the concentration of d-Ala in cells downregulated the expression of genes in peptidoglycan (PG) biosynthesis, including the peptidoglycan-cross-linking transpeptidase genes *ponA* and *dacC*. Consistent with this observation, ultraperformance liquid chromatography-mass spectrometry analysis of murein from P. aeruginosa cells revealed that *dadA* deletion mutants contained PG with reduced cross-linking and altered composition compared to wild-type cells.

## Abstract (precis)

The mechanical properties of bacteria are important for protecting cells against physical stress. The cell wall is the best-characterized cellular element contributing to bacterial cell mechanics; however, the biochemistry underlying its regulation and assembly is still not completely understood. Using a unique high-throughput biophysical assay, we identified genes coding proteins that modulate cell stiffness in the opportunistic human pathogen Pseudomonas aeruginosa. This approach enabled us to discover proteins with roles in a diverse range of biochemical pathways that influence the stiffness of P. aeruginosa cells. We demonstrate that d-Ala—a component of the peptidoglycan—is tightly regulated in cells and that its accumulation reduces expression of machinery that cross-links this material and decreases cell stiffness. This research demonstrates that there is much to learn about mechanical regulation in bacteria, and these studies revealed new nonessential P. aeruginosa targets that may enhance antibacterial chemotherapies or lead to new approaches.

## INTRODUCTION

Bacterial cells live in hostile environments and require physical protection for their survival. For example, turgor pressure within cells reaches 5 to 6 atm for Escherichia coli and 20 to 25 atm for Staphylococcus aureus and changes over short time scales (seconds to minutes) as the molecular composition of extracellular environments fluctuates (1, 2). Bacterial cells live in rapidly flowing fluids, in the corrosive environments of digestive organs, and within deep thermal vents (>350°C); survive the pressure and peristalsis of blood capillaries and arteries; and endure cycles of freezing and thawing (3–7). A stiff cell wall (Young’s modulus of ~25 to 100 mPa [8]) is a key structure for surviving many of these conditions and a hallmark of most bacterial genera; exceptions include mycoplasmas and l-forms (9). The peptidoglycan (PG) layer of the cell wall forms an exoskeleton-like structure that protects cells and is the canonical example of stiff materials in bacteria. With very few exceptions, almost everything known about the chemical and biological elements of bacteria that contribute to cell stiffness connects back to the peptidoglycan layer within the cell envelope and to changes in its structure (10–12).

**FIG 1:**
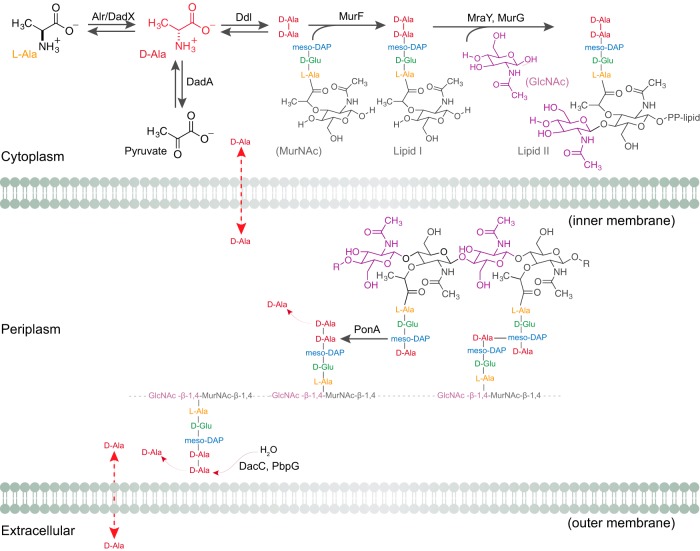
Biochemistry of d-Ala in Gram-negative bacteria. The cartoon represents the utilization and role of d-Ala in bacterial cells. P. aeruginosa cells have two alanine racemases (Alr and DadX) that interconvert l-Ala and d-Ala. DadA is a d-amino-acid dehydrogenase that degrades d-Ala into pyruvate. Ddl is an amino acid ligase that converts two d-Ala molecules into d-Ala-d-Ala, which is a substrate of the enzyme MurF in forming lipid I from the MurNAc tripeptide. MraY and MurG form lipid II, which is subsequently flipped across the membrane into the periplasm and incorporated into the growing peptidoglycan. The PonA transpeptidase cross-links stem peptides during peptidoglycan biosynthesis by releasing the terminal d-Ala into the periplasm. dd-Carboxypeptidase (DacC) and dd-endopeptidases (PbpG) also release the terminal d-Ala from the un-cross-linked lipid II in the periplasm. Free d-Ala in the periplasm and in the extracellular environment is transported into cells through alanine transporters and permeases. “PP-lipid” refers to a diphosphate bridge and long, connected hydrocarbon tail that is attached to the disaccharide in lipid II.

The peptidoglycan consists of linear polysaccharide chains—composed of alternating N-acetylglucosamine (GlcNAc) and N-acetylmuramic acid (MurNAc) units—cross-linked by short peptides (Fig. 1). A d-lactoyl group positioned at the C-3 position on each MurNAc residue is attached to a stem peptide with the common amino acid sequence l-Ala-d-Glu-meso-Dap (or l-Lys-d-Ala-d-Ala); “meso-Dap” refers to meso-diaminopimelic acid (13, 14). Two d-Ala residues at the fourth and fifth positions are universal features of the peptide stem of uncrosslinked peptidoglycan (13, 14). The terminal d-Ala is cleaved off after peptides are cross-linked and is transported into the cell and recycled (15). d-Ala is the most abundant d-amino acid in bacteria and is exclusively incorporated into the peptidoglycan (15). d-Amino acids are generally resistant to enzymatic processing, which presumably protects the peptidoglycan from degradation by proteases with broad-spectrum activity (16).

During peptidoglycan biosynthesis, glycosyltransferases polymerize glycan chains and dd-transpeptidases cross-link stem peptides. Penicillin-binding proteins (PBPs) are a family of enzymes that assemble the peptidoglycan and include enzymes with both glycosyltransferase and transpeptidase activities (class A PBPs) and those with only transpeptidase activity (class B PBPs). Cross-linking of the glycan strands in peptidoglycan generally occurs between the carboxyl group of d-Ala at position 4 of the stem peptide and the amino group of the meso-Dap on the peptide of an adjacent glycan strand; formation of this bond is accompanied by the release of the terminal d-Ala unit (13, 14). Although many of the enzymes involved in peptidoglycan biosynthesis have been identified in the most commonly studied model bacteria, characterization of their function in cells is still in progress. For example, multiple studies, including a study employing a recently introduced genome-wide method for studying bacterial stiffness, demonstrated that PBP1a and PBP1b share biochemical features and yet make remarkably different contributions to cell stiffness (17–22). Thus, there is still much to be learned regarding peptidoglycan biosynthesis in bacterial cells (14, 23).

Antibiotics targeting components of the peptidoglycan biosynthesis machinery disrupt the mechanical integrity of the cell and promote lysis. For example, fosfomycin inhibits biosynthesis of the cell wall precursor lipid II and vancomycin, ramoplanin, and teicoplanin inhibit transglycosylase activity during peptidoglycan chain elongation (1, 10, 24, 25). Amoxicillin, ampicillin, penicillin-G, faropenem, cefixime, and aztreonam prevent amide bond formation between adjacent pentapeptides in peptidoglycan by inhibiting the transpeptidase activity of various PBPs and cause cell lysis (10, 25–29). As peptidoglycan is one of the most important targets for antibiotics, uncovering its biochemical regulation and physical properties (e.g., its contribution to cell stiffness) is potentially important for clinical microbiology and medicine. A general understanding of bacterial cell mechanics is still lacking and would benefit from the following information: (i) identification of the regulators and factors that control peptidoglycan biosynthesis; (ii) a mechanistic understanding of where and when cell wall assembly occurs in cells; and (iii) identification and study of the proteins and materials—including those extending beyond the peptidoglycan—that contribute to cell stiffness (13, 30–37).

Very few bacterial species have been the focus of cell mechanics studies to date (12, 38, 39). Among those that have not been mechanically characterized, Pseudomonas aeruginosa is a model Gram-negative bacterium that is clinically relevant and difficult to treat using chemotherapies (40–42). P. aeruginosa is notoriously resistant to different families of antibiotics, showing low susceptibility to beta-lactams, aminoglycosides, and quinolones (40, 41). The genomes of multiple P. aeruginosa strains have been sequenced; however, only 20% of the 5,973 predicted genes in P. aeruginosa strain PA14 have an assigned function (43), making it an interesting organism in which to build connections between genes and cell stiffness. We envisioned that research on P. aeruginosa cell stiffness may continue to close the biochemical gap among genes of unknown function and potentially reveal new targets for developing antibiotic strategies.

Here we performed a genome-wide cell mechanics screen in P. aeruginosa to identify and study the biochemical elements that control cell mechanics and that may be exploited to alter cell mechanical properties synthetically. By screening a nonredundant transposon library of gene knockouts in P. aeruginosa strain PA14, we identified 42 candidate proteins that significantly altered cell mechanics (44). Our studies illuminate the importance of d-Ala catabolism in regulating cell stiffness, its effect on transcriptional regulation of enzymes that cross-link peptidoglycan, and the downstream changes in cell stiffness arising from a decrease in peptidoglycan cross-linking. The results of these studies suggest the presence of an uncharacterized biochemical network in cells and a potential target to exploit in chemotherapies against P. aeruginosa.

## RESULTS AND DISCUSSION

### Amino acid metabolism genes are enriched in a genome-wide screen of P. aeruginosa PA14 cell stiffness.

We used a previously characterized high-throughput assay (general regulators affecting bacterial stiffness [GRABS] [17]) to identify P. aeruginosa cells with altered stiffness from a library of transposon mutants. Specifically, we screened a nonredundant transposon mutant library of strains of P. aeruginosa PA14 consisting of 5,790 clones covering ~80% of the nonessential PA14 open reading frames (ORFs) to identify mutants with altered cell stiffness (44). Liberati et al. used a mariner-based transposon, MAR2xT7, to construct mutants in which a gentamicin resistance gene was inserted into random loci in the P. aeruginosa genome (44). We measured the growth of cells of each mutant strain against that of wild-type P. aeruginosa PA14 in liquid lysogeny broth (LB) nutrient media and encapsulated in 1% agarose gels infused with LB. We normalized the absorbance value (λ = 595 nm) of wild-type PA14 cells grown in liquid LB and in 1% agarose and used these data to determine a percent growth value for each mutant compared to wild-type cells. We determined a relative stiffness score—referred to here as a GRABS score (17)—for all mutant strains against wild-type PA14 using the following equation (where “OD” represents optical density [i.e., absorbance]):

$$\text{GRABS score}=100\;\times \left(\frac{{\text{OD}}_{\text{mutant, agarose}}}{{\text{OD}}_{\text{wild type, agarose}}}-\frac{{\text{OD}}_{\text{mutant, LB}}}{{\text{OD}}_{\text{wild type, LB}}}\right)$$

For wild-type and mutant cells growing at the same rate in liquid LB, a positive GRABS score indicates a mutant growing faster in 1% agarose than wild-type cells. On the basis of our past experiments, mutants with a positive GRABS score represent increased stiffness compared to wild-type cells. A negative GRABS score for a mutant indicates cells with reduced stiffness and growth in 1% agarose that is slower than that of wild-type cells (again, when the growth rates of both wild-type and mutant cells in liquid LB are similar). P. aeruginosa PA14 mutants with high or low scores revealed genes coding for proteins that are likely to play a role in cell stiffness.

**FIG 2:**
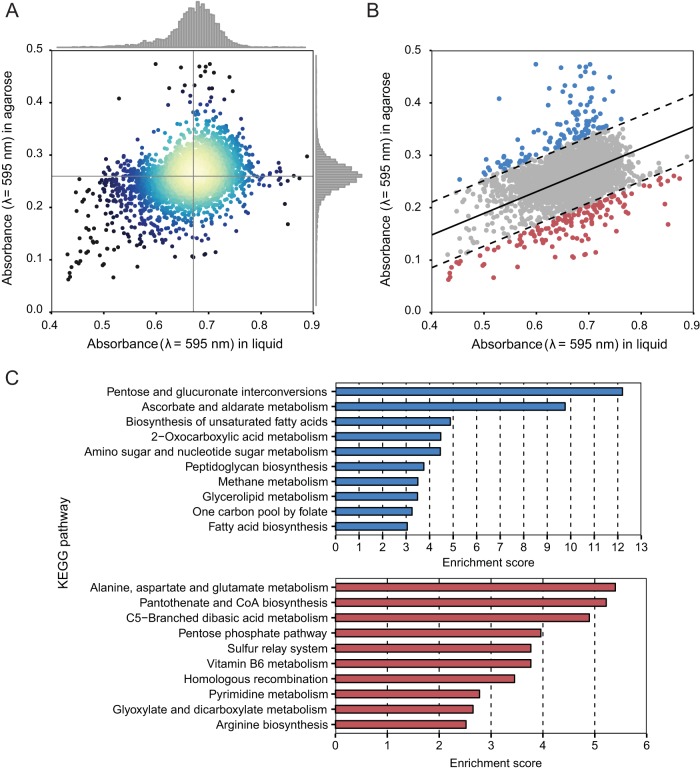
Genome-wide stiffness screen of Pseudomonas aeruginosa. (A) A scatter plot of all gene transposon mutants in P. aeruginosa PA14 and corresponding absorbance values (λ = 595 nm) for cell growth in LB and in 1% agarose infused with LB; each point represents a single gene transposon mutant. Regions in the plot with the highest density of data points are depicted in yellow. (B) A plot of genome-wide stiffness screening data fitted using a bivariate normal distribution. Transposon mutants highlighted by blue data points (*n* = 115 genes) had higher growth in 1% agarose than the mutants highlighted by red data points (*n* = 133 genes). Genes that lie within the interval between two dashed lines (in gray) followed a linear growth model. (C) A summary of KEGG pathway enrichment for the data depicted in blue (in panel B) with higher cell stiffness and the data depicted in red with lower cell stiffness.

Figure 2A displays a scatter plot of every gene in the library and time-dependent absorbance measurements for mutants growing in LB (λ = 595 nm) and for mutants that were encapsulated and growing in 1% agarose infused with LB. From an analysis of the distribution of absorbance measurements (λ = 595 nm) of mutants in LB and in agarose, we found that absorbance was approximately normally distributed and centered at values of 0.67 in LB and 0.27 in 1% agarose. Of the 5,728 mutants that we studied, 35 mutants did not grow in LB and 1% agarose and were excluded from our analysis, which reduced the number of genes in the data set to 5,693. Using two variables—the absorbance (λ = 595 nm) in LB and 1% agarose—we fitted a bivariate normal distribution to determine the direction of maximum variance in the data set represented by a straight line. This line represents a positive, linear correlation between cell growth in 1% agarose and cell growth in liquid LB media; the majority of the 5,445 mutants were positioned within 3 standard deviations from this line, indicating a strong correlation between the levels of P. aeruginosa cell growth in 1% agarose and in LB (Fig. 2B). We calculated the geometric distance of the data from this line for every gene and filtered mutants whose data were positioned 3 standard deviations away from the line. These mutants had a minimum correlation between their absorbance measurements in 1% agarose and in liquid LB and lay farthest from the line of maximum variance within the data set. Most of these genes had a large increase or decrease of cell growth in 1% agarose, and yet they maintained a growth rate in liquid LB similar to that of P. aeruginosa wild-type PA14 cells. P. aeruginosa mutants (depicted by blue data points in Fig. 2B; *n* = 115) had higher absorbance (λ = 595 nm) in 1% agarose than the mutants depicted by red data points (*n* = 133), suggesting that mutants the highlighted in red had lower stiffness values (Fig. 2B). We performed gene enrichment analyses of mutants from both the blue and red data sets using Fisher’s exact test to correlate KEGG pathways with altered cell stiffness in our assay. A variety of KEGG pathways were enriched in mutants with higher stiffness (blue data points), including those that correlated to peptidoglycan biosynthesis and glycerolipid metabolism pathways. KEGG pathways enriched for lower-stiffness mutants (red data points) included alanine, aspartate, and glutamate metabolism pathways (Fig. 2C).

**FIG 3:**
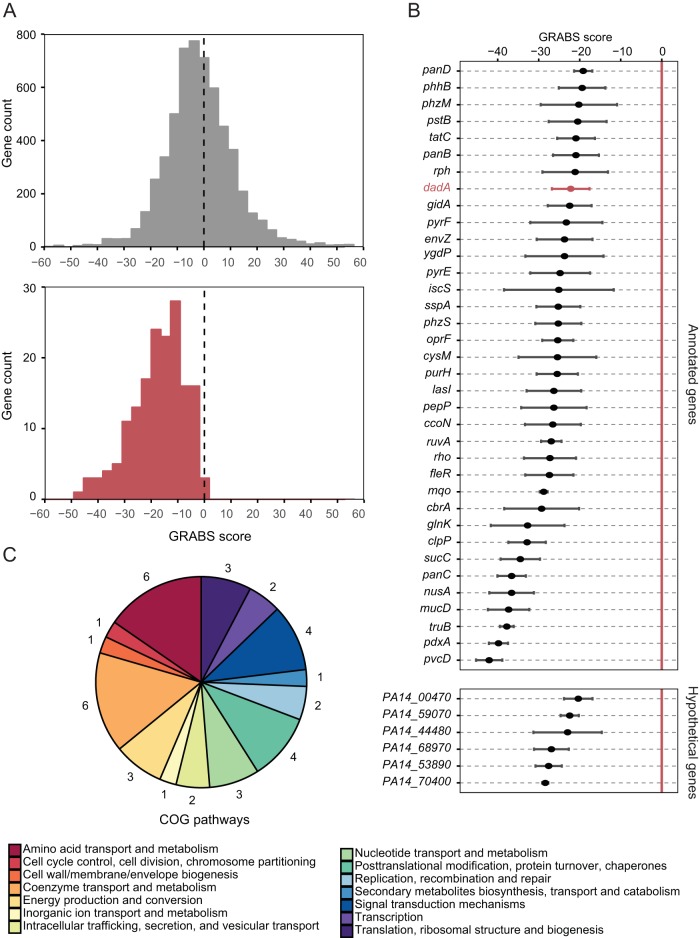
Stiffness genes code proteins involved in diverse biochemical pathways. (A) A histogram depicting that the GRABS score distribution of rescreened genes (bottom panel) shows a reduction in the mean GRABS score compared to the genes in the entire screen (top panel). (B) A plot of the GRABS scores along with the corresponding variance for 42 rescreened genes. 36 out of 42 genes were annotated and had an assigned biochemical function. 6 of the top 42 hits are not yet annotated and are named after their respective gene locus. The P. aeruginosa PA14 *dadA*::Tn mutant (depicted in a red) has very low variance in the GRABS score and consistently produces a negative GRABS score. (C) Gene ontology information (COG, classification of gene ontology) for the top hits. The numbers surrounding the pie chart indicate the number of genes (out of the 42 selected) that are represented within each COGs family.

We determined GRABS scores for all mutants in the P. aeruginosa PA14 library to qualitatively compare mechanical changes and found the distribution of scores to be approximately normal (Fig. 3A). We rescreened 178 mutants from the whole-genome screen that had GRABS scores of less than −10 to confirm the results. The rescreened mutants had predominately negative GRABS scores, and the distribution of values was shifted toward mean GRABS values (−17.5) that were lower than those determined from screening the entire mutant collection (Fig. 3A). Rescreening of the top hits enabled us to assign variance for individual genes with a negative GRABS score. A list of 42 mutants with the largest negative GRABS scores (i.e., those with scores of −20) is illustrated in Fig. 3B (Table S1). Six mutants did not have an assigned function, suggesting that the proteins coded by these genes may play a role in cell mechanics.

10.1128/mBio.01340-18.9 TABLE S1  List of 42 genes with largest negative GRABS score related to Fig. 3. Download TABLE S1, XLSX file, 0.04 MB. Copyright © 2018 Trivedi et al. 2018 Trivedi et al. This content is distributed under the terms of the Creative Commons Attribution 4.0 International license.

We were interested in the cellular distribution of proteins encoded by stiffness-related genes across different cellular compartments in P. aeruginosa, namely, those that are cytoplasmic, periplasmic, extracellular, and associated with each membrane, and we performed a subcellular localization analysis of the Pseudomonas Genome Database (http://www.pseudomonas.com) (45). Our analysis of the 36 mutants with the largest negative GRABS scores identified 24 cytoplasmic proteins, 7 proteins associated with the cytoplasmic membrane, 2 periplasmic proteins, and 1 protein associated with the outer membrane; no information regarding subcellular localization was available for 2 proteins (see Fig. S1 in the supplemental material).

10.1128/mBio.01340-18.1 FIG S1  Subcellular localization of 36 genes with the largest negative GRABS scores. Download FIG S1, PDF file, 0.2 MB. Copyright © 2018 Trivedi et al. 2018 Trivedi et al. This content is distributed under the terms of the Creative Commons Attribution 4.0 International license.

We performed a gene ontology analysis of the cluster of orthologous groups (COGs) corresponding to mutants with the largest change in cell stiffness and grouped them based on these categories (Fig. 3C). We also compared the results of a COGs analysis of the stiffness regulators from P. aeruginosa to those determined for the stiffness regulators from E. coli. Although there were no individual homologues shared between these two species of bacteria, grouping these genes based on COGs, we found significant overlap. We found several COGs that were well represented and that contained at least 3 representatives in each of the following categories: energy production and conversion, amino acid metabolism, nucleotide metabolism, and signal transduction.

*dadA* was a gene in the amino acid transport and metabolism COGs family that caught our attention for several reasons: (i) it belongs to the most enriched pathway found in the KEGG gene enrichment analysis (i.e., alanine, aspartate, and glutamate metabolism); (ii) it is clustered in the amino acid transport and metabolism COGS category; and (iii) it codes for a cytoplasmic enzyme involved in the pathway for d-Ala metabolism, connecting it directly to peptidoglycan assembly (Fig. 3B).

### d-Ala dehydrogenase (DadA) is a modulator of P. aeruginosa PA14 cell stiffness.

d-amino acids are generally present in low concentration in cells; d-Ala is the most prevalent of the d-amino acids in bacteria. In bacteria, Ala racemases (Alr) convert l-Ala to d-Ala, which is subsequently incorporated into the peptidoglycan (Fig. 1) (46). Ala racemases have emerged as a well-studied class of enzymes and as drug targets for antibiotics due to the role of d-Ala in the peptidoglycan layer of the bacterial cell wall (47–49). P. aeruginosa contains two alanine racemases: Alr and DadX. Mutagenesis studies have revealed low levels of constitutively expressed Alr that serve an anabolic function in peptidoglycan assembly (46). In contrast, DadX converts l-Ala to d-Ala in the alanine catabolism pathway (48). DadA is a d-Ala dehydrogenase that oxidatively deaminates d-Ala to pyruvate and ammonia (Fig. 1) (50). Collectively, DadA, DadX, and Alr play an essential role in the utilization of d-Ala and l-Ala as sources of carbon and energy for cell growth (48, 51).

During peptidoglycan synthesis, d-Ala-d-Ala ligases (Ddl) consume the cytoplasmic pool of d-Ala to form d-Ala-d-Ala dipeptides, which are ligated on the tripeptide stem of UDP-MurNAc to form the UDP-MurNAc pentapeptide (Fig. 1) (23). MurG catalyzes the addition of GlcNAc to lipid I (~700 copies/cell) to produce lipid II (~2,000 copies/cell)—the building block of peptidoglycan—containing the complete disaccharide pentapeptide monomer unit (52). Lipid II is transported across the cytoplasmic membrane into the periplasm, where it is incorporated into peptidoglycan (14, 52). d-Ala is also released by transpeptidase and carboxypeptidase activities involved in peptidoglycan assembly and remodeling. During cross-linking of newly synthesized peptidoglycan strands, transpeptidases cleave the terminal d-Ala and transporters move it from the periplasm to the cytoplasm (Fig. 1) (13, 14, 53).

**FIG 4:**
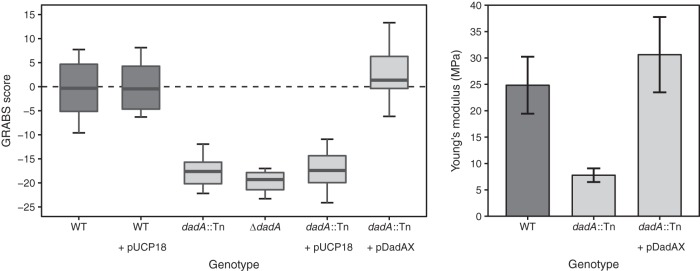
d-Ala dehydrogenase (DadA) is a modulator of P. aeruginosa cell stiffness. (Left panel) GRABS score for P. aeruginosa wild-type cells, *dadA*::Tn cells, and the *dadA* complementation strain. (Right panel) Young’s modulus of wild-type cells, *dadA*::Tn cells, and the *dadA* complementation strain. Stiffness measurements were performed using a microfluidic cell-bending assay.

We studied the role of DadA in P. aeruginosa cell stiffness. The transposon mutant of *dadA* in P. aeruginosa PA14 (*dadA*::Tn) had a GRABS score of −18 (Fig. 4, left panel). We also constructed an in-frame *dadA* deletion strain, mutant Δ(*dadA*), and determined a GRABS score of −20. As the Δ(*dadA*) strain and strain *dadA*::Tn had similar GRABS scores, we used the transposon deletion mutation throughout the study.

To confirm that the change in cell stiffness was the result of deleting *dadA*, we complemented cells by expressing *dadA* in the PA14 *dadA*::Tn strain. Complementation of the *dadA* mutant requires expression of both *dadA* and *dadX* due to polar effects of the *dadA* MAR2xT7 transposon insertion (48). We followed this precedent by complementing *dadA* with *dadX* using vector pUCP18 as reported previously (48). Overexpressing DadA and DadX in Δ*dadA* cells of P. aeruginosa PA14 led to a complete recovery of the growth phenotype in 1% agarose and rescued the GRABS phenotype for the *dadA* transposon mutant (Fig. 4, left panel). To test whether sole expression of DadX would affect the GRABS score, we determined the GRABS score of *dadX*::Tn (score, −0.7), *dadX*::Tn expressing empty vector (score, 1.1), and *dadX*::Tn overexpressing DadX (score, −1.2). Failing to observe any stiffness defect for *dadX*::Tn or the *dadX*::Tn strain overexpressing DadX, we conclude that DadX does not contribute to the stiffness (Fig. S2).

10.1128/mBio.01340-18.2 FIG S2  DadX did not contribute to cell stiffness. Download FIG S2, PDF file, 0.2 MB. Copyright © 2018 Trivedi et al. 2018 Trivedi et al. This content is distributed under the terms of the Creative Commons Attribution 4.0 International license.

To confirm that the P. aeruginosa PA14 *dadA*::Tn mutant has reduced cell stiffness, we used a microfluidic approach to measure the bending rigidity of P. aeruginosa PA14 cells, including wild-type cells, *dadA*::Tn cells, and *dadA*::Tn cells containing p*dadAX*. In bending assays, we applied a shear fluid force in a central channel to dozens of filamentous cells loaded into the side channels, which resulted in horizontal deflection of the cell tips along the direction of the fluid flow. Fitting the data on cell deflection to a mechanical model provided us with accurate values of the (flexural) bending rigidity of cells (17, 54). This approach provides an advantage over other mechanical measurements (e.g., atomic force microscopy [AFM]) as it measures the composite modulus across intact, living cells. Using a thickness of 3.0 ± 0.5 nm for the peptidoglycan layer of P. aeruginosa (55), we converted values of bending rigidity to values representing Young’s modulus (*E*; the intrinsic stiffness of a material defined by the slope of the stress strain curve) and found *dadA* transposon mutant cells to have a 3-fold reduction in Young’s modulus (*E* = 7.8 MPa) compared to wild-type cells (*E* = 25 MPa) (Fig. 4, right panel). Complementation by DadA and DadX in the *dadA*::Tn mutant rescued the stiffness phenotype (*E* = 30 MPa). These results suggest that *dadA* contributes significantly to P. aeruginosa cell stiffness, leading to the hypothesis that inhibiting the d-Ala degradation pathway increases the cellular concentration of free d-Ala and reduces cell stiffness by an unknown mechanism.

### Increases in d-Ala levels reduce cell stiffness.

DadA is involved in catabolism of cytosolic d-Ala and its conversion into pyruvate. We hypothesized that the transposon mutant of *dadA* may increase the concentration of cytosolic d-Ala or decrease the concentration of pyruvate or do both. Pyruvate in Gram-negative bacteria is predominantly produced by glycolysis, and the intracellular concentration of pyruvate is 390 µM (56), which is estimated to be 100× higher than the combined concentrations of d-Ala and l-Ala, i.e., 4 µM (57). When pyruvate levels are kept approximately constant, catabolism of d-Ala contributes <1% to the intracellular pool of pyruvate, suggesting that this pathway is unlikely to be responsible for changing the levels of cell stiffness and that the accumulation of d-Ala may instead be responsible.

**FIG 5:**
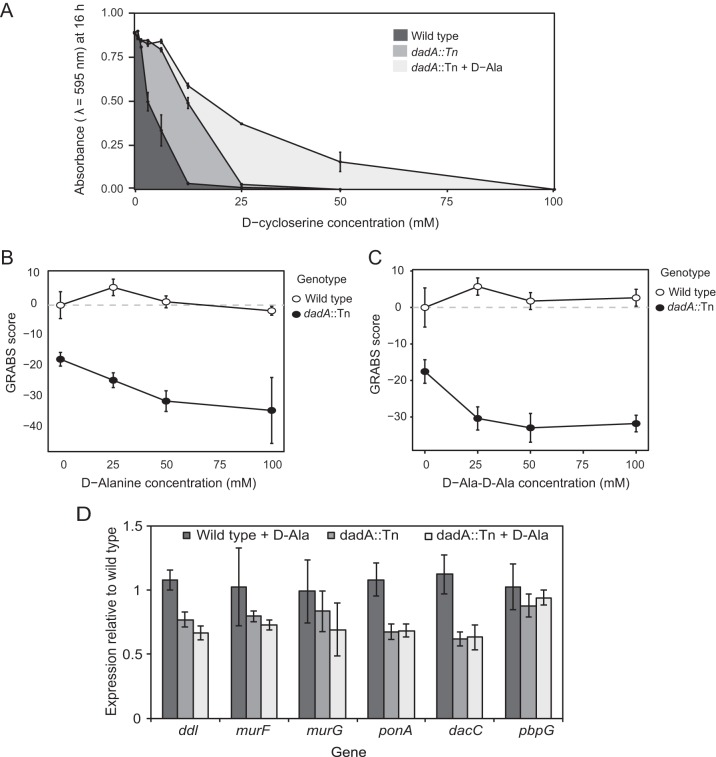
The peptidoglycan biosynthetic pathway is sensitive to d-Ala levels. (A) P. aeruginosa PA14 *dadA*::Tn mutant cells grew better than the PA14 wild-type strain in the presence of a sub-MIC level of DCS. *y*-axis data represents absorbance (λ = 595 nm) after 16 h of growth. Adding d-Ala to the growth media enhanced the growth phenotype of the *dadA*::Tn mutant. (B) An increase in the concentration of exogenous d-Ala (in the growth media) reduced the GRABS score for *dadA*::Tn mutant cells compared to wild-type cells. (C) *dadA*::Tn mutant cells grown in LB media supplemented with d-Ala-d-Ala (15 mM) had a 30% decrease in the GRABS score compared to wild-type cells. (D) Transcription of *ponA*, *dacC*, *murF*, and *ddl* was reduced in P. aeruginosa PA14 *dadA*::Tn mutant cells compared to wild-type cells. These genes code for proteins that either release d-Ala into the periplasm (*dacC* and *ponA*) or utilize d-Ala as a substrate for peptidoglycan precursor synthesis (*ddl* and *murF*).

To explore the hypothesis that an increase in the intracellular concentration of d-Ala reduces P. aeruginosa cell stiffness and to confirm that the *dadA*::Tn mutant cells had a higher concentration of d-Ala than the P. aeruginosa PA14 wild-type cells, we treated cells with d-cycloserine (i.e., DCS; 4-amino-3-isoxazolidinone). DCS is a cyclic mimic of d-Ala that inhibits Ala racemases (Alr) and d-Ala-d-Ala ligases (Ddl) and inhibits cell growth by decreasing the intracellular pool of d-Ala (58–60). The MIC of DCS against P. aeruginosa PA14 wild-type cells was 2-fold lower (12 mM) than that seen with the *dadA* transposon mutants (25 mM). Both the wild-type strain and the *dadA* transposon mutants were grown in the absence of gentamicin to measure the MIC of DCS. We measured the growth of the P. aeruginosa PA14 wild-type strain and *dadA* transposon mutants in the presence of various DCS concentrations and observed that the *dadA*::Tn mutant grew to an absorbance of 0.5 (λ = 595 nm) whereas the wild-type cells grew to an absorbance of only 0.03 after 16 h of incubation, suggesting that an increase in the intracellular pool of d-Ala in the *dadA* mutant suppresses growth inhibition by DCS (Fig. 5A). Supplementing growth media with d-Ala (25 mM) enabled the *dadA*::Tn mutant to tolerate 4× the MIC of DCS (50 mM) and to grow to an absorbance of 0.15 (λ = 595 nm) after 16 h. These data are consistent with a higher intracellular level of d-Ala in the *dadA* transposon mutant titrating down the inhibitory effect of DCS on cell growth (Fig. 5A). In a control experiment, we also supplemented the media with l-Ala (25 mM) and measured the MIC of DCS against cells of the wild-type strain and the *dadA*::Tn mutant. Unlike the results seen with d-Ala supplementation, the wild-type and *dadA*::Tn mutant strains had similar sensitivities to DCS; the MIC was 25 mM in the presence of l-Ala (Fig. S3). The MICs for DCS against P. aeruginosa are presumably high due to the extensive drug efflux system present; in contrast, DCS has been measured to have MICs in the range of 50 to 500 µM in Mycobacterium smegmatis and Escherichia coli (61).

10.1128/mBio.01340-18.3 FIG S3  In the presence of l-Ala, *dadA*::Tn and wild-type cells had similar sensitivities against DCS. Download FIG S3, PDF file, 0.2 MB. Copyright © 2018 Trivedi et al. 2018 Trivedi et al. This content is distributed under the terms of the Creative Commons Attribution 4.0 International license.

If a reduction in cell stiffness were linked to an increase in the d-Ala concentration, we would expect P. aeruginosa cells grown in the presence of exogenous d-Ala to have a lower GRABS score than cells growing in the absence of d-Ala. P. aeruginosa has at least two mechanisms for transporting d-Ala into cells: (i) by diffusion through porin channels or by active, carrier-mediated proton motive force-dependent systems (53, 62, 63) and (ii) by the activity of amino acid permeases (53). Taking advantage of these systems to test the effect of the d-Ala concentration on the stiffness of P. aeruginosa PA14 cells, we grew cells in the presence of nutrient media containing different d-Ala concentrations (25, 50 and 100 mM) and determined the GRABS scores for wild-type and *dadA*::Tn mutant cells. Growing cells of the *dadA*::Tn mutant in the presence of 25 mM d-Ala decreased the GRABS score to −24 compared to the GRABS score of −18 for the *dadA*::Tn mutant grown in the absence of d-Ala. Increasing the d-Ala concentration to 50 mM reduced the GRABS score to −31; at a concentration of 100 mM d-Ala, the GRABS score was −34 (Fig. 5B). P. aeruginosa PA14 wild-type cells grown in the presence of 25 to 100 mM d-Ala showed no change in the GRABS score (Fig. 5B). These results suggest that wild-type cells regulate the concentration of d-Ala in cells and that increasing the d-Ala concentration reduces cell stiffness. We also compared the GRABS score of *dadA*::Tn cells grown in the presence of l-Ala with that of the cells grown in LB lacking l-Ala. Growing cells in the presence of l-Ala did not alter the stiffness of the mutant, and the GRABS score was −19, which is similar to the GRABS score of −18 determined with plain LB (Fig. S4).

10.1128/mBio.01340-18.4 FIG S4  The *dadA*::Tn mutant GRABS score did not change when the mutant was grown in the presence of l-Ala. Download FIG S4, PDF file, 0.2 MB. Copyright © 2018 Trivedi et al. 2018 Trivedi et al. This content is distributed under the terms of the Creative Commons Attribution 4.0 International license.

We measured the transcription of *dadA* in response to the concentration of d-Ala levels in growth media. Changes in *dadA* transcription in P. aeruginosa PA14 were determined by performing quantitative PCR (qPCR) on cells growing under two conditions: (i) LB and (ii) LB supplemented with 25 mM d-Ala. We found that P. aeruginosa PA14 cells grown in the presence of 25 mM d-Ala have a 2-to-3-fold-higher level of *dadA* transcription than cells grown in LB (Fig. S5). These results are consistent with previously published measurements of *dadA* promoter activity in P. aeruginosa strain PAO1; the level was 12-fold higher for cells grown in the presence of d-Ala than for cells grown in nutrient media without d-Ala (51). P. aeruginosa cells thus appear to homeostatically regulate the intracellular concentration of d-Ala (e.g., through DadA).

10.1128/mBio.01340-18.5 FIG S5  Wild-type P. aeruginosa PA14 cells had higher *dadA* transcription when the LB media were supplemented with 25 mM d-Ala. Download FIG S5, PDF file, 0.2 MB. Copyright © 2018 Trivedi et al. 2018 Trivedi et al. This content is distributed under the terms of the Creative Commons Attribution 4.0 International license.

We hypothesized that the stiffness phenotype that we observed for d-Ala and the *dadA* transposon mutant is due to an increase in the intracellular concentration of the d-Ala-d-Ala dipeptide—one of two substrates for MurG on the route to synthesis of lipid II—altering peptidoglycan structure or assembly. To test this hypothesis, we determined the GRABS scores for PA14 wild-type cells and *dadA*::Tn mutant cells in LB supplemented with different concentrations of d-Ala-d-Ala (15, 25, 50, and 100 mM). We observed a 30% decrease in the GRABS score for the *dadA*::Tn mutant in the presence of 15 mM d-Ala-d-Ala; higher d-Ala-d-Ala concentrations did not reduce mutant GRABS scores further (Fig. 5C). We also measured the Young’s modulus for P. aeruginosa PA14 wild-type and *dadA*::Tn cells grown in d-Ala (100 mM) and d-Ala-d-Ala (100 mM) using the microfluidics-based bending assay. The Young’s modulus of the *dadA*::Tn strain grown in d-Ala (*E* = 7.0 MPa) or d-Ala-d-Ala (*E* = 6.1 MPa) was lower than that determined for the mutant grown in LB (*E* = 7.8 MPa) (Fig. S6); however, the magnitudes of the changes in our microfluidic bending data were not closely correlated to the results of the GRABS assays for reasons unknown.

10.1128/mBio.01340-18.6 FIG S6  *dadA*::Tn had a lower Young’s modulus value when the growth media were supplemented with d-Ala or d-Ala-d-Ala. Download FIG S6, PDF file, 0.2 MB. Copyright © 2018 Trivedi et al. 2018 Trivedi et al. This content is distributed under the terms of the Creative Commons Attribution 4.0 International license.

### Peptidoglycan synthesis is sensitive to the concentration of d-Ala.

Although the hypothesis is untested, high d-Ala concentrations may affect the transcription level of enzymes that biosynthesize peptidoglycan, alter the structure or composition of peptidoglycan, and regulate the mechanical properties of P. aeruginosa cells. We used qPCR to measure the transcription of various genes coding for proteins involved in different stages along the peptidoglycan biosynthetic pathway. Specifically, we selected diverse genes involved in peptidoglycan biosynthesis coding for amino acid ligases (Ddl and MurF), a glycosyltransferase (MurG), a transpeptidase (PonA), a dd-carboxypeptidase (DacC), and a dd-endopeptidase (PbpG) (Fig. 1).

A comparison of the transcription levels of amino acid ligases encoded by *ddl* and *murF* in the P. aeruginosa PA14 wild-type strain and the *dadA*::Tn mutant indicated that *ddl* and *murF* were downregulated 23% and 20%, respectively, in the *dadA* transposon mutant compared to wild-type cells growing in LB. When we supplemented LB medium with 25 mM d-Ala, we observed that *ddl* and *murF* were further downregulated 33% and 27%, respectively, in the *dadA*::Tn mutant whereas PA14 wild-type cells did not show any changes in the levels of transcription of these genes. Ddl ligates two d-Ala molecules into the d-Ala-d-Ala dipeptide, and its activity is strongly inhibited by its product d-Ala-d-Ala (64–66), suggesting the presence of a feedback mechanism to regulate its intracellular concentration. Consequently, cells may use multiple mechanisms to counter high concentrations of d-Ala, such as increasing *dadA* transcription and downregulating expression of Ddl to control levels of d-Ala-d-Ala in cells (Fig. 1).

MurG is a glycosyltransferase that produces lipid II by transferring UDP-GlcNac to lipid I (Fig. 1). We did not observe a significant difference in the transcription levels of *murG* in P. aeruginosa PA14 wild-type and *dadA*::Tn mutant cells in the presence or absence of d-Ala in LB. As subsequent steps in peptidoglycan biosynthesis occur in the periplasmic space after lipid II is translocated to the periplasm, we explored changes in the transcription levels of genes encoding proteins that participate in peptidoglycan biosynthesis in the periplasm. The P. aeruginosa PonA transpeptidase cross-links the peptide chains between lipid II and the peptidoglycan; we observed a 32% reduction in *ponA* transcription in PA14 *dadA*::Tn mutant cells (Fig. 5D), which may reduce peptidoglycan cross-linking and cause a downstream decrease in cell stiffness in the absence of DadA. Further support for the idea of changes in cross-linking in *dadA*::Tn mutant cells comes from experiments performed on dd-carboxypeptidases, which hydrolyze the terminal d-Ala from the pentapeptide stem of the nascent peptidoglycan and render the peptide unavailable for transpeptidation reactions (67, 68). We quantified the transcription level of *dacC*, one of the most abundant dd-carboxypeptidases in Gram-negative bacteria, and observed a 38% reduction in the absence of DadA, supporting the hypothesis that a decrease in peptidoglycan cross-linking in the *dadA*::Tn mutant may be responsible for the decrease in cell stiffness (Fig. 5D). When we supplemented LB with d-Ala and measured the transcription levels of both *ponA* and *dacC* in the *dadA* mutant, we did not observe any significant difference, suggesting a basal level of transpeptidase and carboxypeptidase activity. We also measured changes in transcription of *pbpG*, encoding a dd-endopeptidase, in wild-type and *dadA*::Tn mutant cells. PbpG hydrolyzes the peptide bond between side chains on two glycan strands in peptidoglycan, thereby countering the activity of dd-transpeptidases. We did not observe a change in the level of *pbpG* transcription in *dadA*::Tn mutant cells (Fig. 5D), suggesting that the mutants have a level of endopeptidase activity that may match that of wild-type cells.

**FIG 6:**
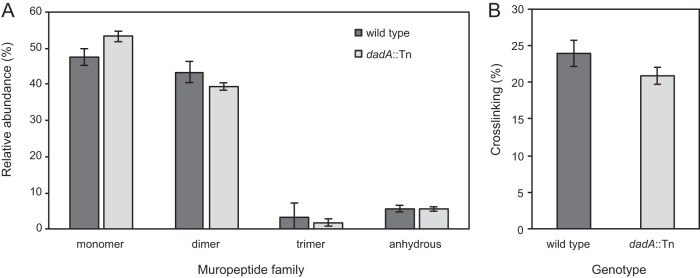
The peptidoglycan composition of *dadA*::Tn mutant cells is altered compared to P. aeruginosa wild-type cells. (A) UPLC-MS data revealed that the muropeptide composition of the P. aeruginosa wild-type strain and that of the *dadA* mutant differ in the abundance of monomer, dimer, and anhydrously terminated saccharides. *n* = 3 biological replicates. (B) We observed a decrease in peptidoglycan cross-linking of *dadA*::Tn compared to wild-type cells. Error bars represent standard deviations of the means.

Our results demonstrate that regulation of d-Ala levels in P. aeruginosa is wired to the transcription of genes coding for proteins that are pivotal in cross-linking the peptidoglycan. Transpeptidase reactions are reversible and sensitive to the concentration of d-Ala (69); a decrease in cross-linking is presumably responsible for changes in cell stiffness of the DadA mutant, which has a higher concentration of intracellular d-Ala. We used ultraperformance liquid chromatography-mass spectrometry (UPLC-MS) to quantify differences in peptidoglycan composition and cross-linking density in DadA mutant and wild-type cells (70). *dadA*::Tn mutant cells contained a higher concentration of monomers and fewer dimers and trimers than wild-type cells and showed virtually no difference in the levels of anhydrous-containing saccharides (which are chain terminators in polymerizing saccharides) (Fig. 6A). We calculated the PG cross-linking densities (71) of wild-type and *dadA*::Tn cells and found the level of density to be reduced by 12.5% in the mutant (Fig. 6B). Although we did not observe large differences in peptidoglycan composition and cross-linking between these cell types, the field still lacks a relationship to correlate the magnitude of cell stiffness and peptidoglycan cross-linking. Our results are consistent with the hypothesis that the concentration of d-Ala is tightly regulated in P. aeruginosa cells and is connected to the regulation of peptidoglycan cross-linking and that an increase in the concentration of d-Ala reduces peptidoglycan cross-linking and cell stiffness.

In summary, using the GRABS assay, we identified 42 new cell stiffness regulators in P. aeruginosa by screening a genome-wide collection of transposon mutants from a nonredundant transposon library of P. aeruginosa strain PA14. The genes that we discovered had three interesting characteristics: (i) they were ontologically diverse; (ii) they were historically disconnected from cell mechanics; and (iii) they revealed surprising connections between physiology and cell mechanics (Fig. 3C). We demonstrate one pathway among many hits from the screen and observed that there is still much to learn about how bacteria regulate their mechanical properties. Further experiments will validate DadA and other assay hits as antibiotic targets, ideally in combination with traditional cell wall-targeting antibiotics that have limited activity against clinical strains of Pseudomonas aeruginosa.

## MATERIALS AND METHODS

### Strain and plasmid construction.

Screening was performed using the nonredundant transposon mutant library of P. aeruginosa strain PA14 consisting of 5,790 transposon mutants (44).

### Growth of bacterial cultures for screening.

Individual 2-ml cultures were inoculated from a freezer stock and grown overnight in lysogeny broth (LB) at 37°C with shaking until saturation (~16 h). Since the transposon library contains a gentamicin resistance cassette, mutants were grown in the presence of 15 µg/ml gentamycin for selection of the transposon mutant in the overnight culture. Strains containing plasmid pUCP18 were grown overnight in 100 µg ampicillin or 100 µg gentamycin (48).

### Preparation of GRABS 96-well plates.

To prepare GRABS 96-well plates, we used a previously published GRABS protocol (17). Briefly, absorbance (*λ* = 595 nm) of saturated overnight cultures was measured for different mutant strains and cells were harvested at 1,000 × *g* for 10 min to give a final absorbance value of 0.32. A total of 48 microstirrers (V&P Scientific, San Diego, CA) were washed with 70% ethanol and placed into individual wells of first 6 columns of a 96-well microplate (Corning), and the microplate was sterilized with UV light for 20 min before being transferred to a 50°C hot plate to warm the plate and magnets. To prepare the final plate for growth curve measurements, we pipetted 150 µl of fresh LB (with no antibiotic) into the wells in last 6 columns (columns 7 to 12) and let the LB warm for 20 min. A 1% UltraPure agarose solution was prepared in LB until the solution was visually homogeneous, and 150 µl was added to each well using a positive displacement pipette and stirred for ~10 s. The microplate was transferred to a second hot plate set to 37°C and cooled for 15 to 20 s. We aliquoted 5 µl of each cell suspension into one well with LB and one well with agarose prepolymer using a multichannel pipette to yield an OD of 0.01 and mixed the cultures with magnetic stirrers for ~10 s.

We extracted the magnetic stirrers using another magnet and quickly removed any bubbles on the surface of the agarose gel with a sterile pipette tip. Once the agarose had solidified, we sealed the 96-well GRABS plate with a transparent polymer film. Finally, we placed the microplate into a preheated 37°C M1000 Tecan plate reader and recorded absorbance at 595 nm at 37°C. We used a duration of 30 s for orbital and linear shaking and agitated plates at an amplitude of 2 mm. Readings were taken for 16 h at 5-min intervals, and i-control v. 1.9.17.0 software (Tecan) was used to collect and export data from the plate reader for further analysis.

### GRABS data analysis.

Growth data were collected over 16 h for PA14 transposon mutants in both agarose and liquid media. To reduce the growth curve to a scalar value, we first normalized the growth data in liquid and agarose by subtracting the minimum absorbance reading (in agarose and liquid) for each mutant. We determined the growth saturation point of the P. aeruginosa wild type using a custom MatLab script. The growth curve data were smoothed, and the absorbance of a blank sample was subtracted from all the traces. To smooth the data, we utilized a moving average algorithm with a window size of 20 data points. To determine the time of growth saturation of the bacteria, we analyzed the slope of the smoothed absorbance curve as the curve approached saturation. We introduced a threshold on the slope to determine the first point at which the bacteria reached saturation; the threshold was set at a value of 5 × 10^−6^. If the slope never dipped below this value, we excluded the curve from our analysis. On the basis of the moving average algorithm, we took the average of 7 data points at 12 h, which is approximately when PA14 wild-type cells begin to enter stationary phase in liquid medium. We also used 12-h time points to calculate absorbance for cells embedded in 1% agarose, as that provided the largest dynamic range of values to compare.

We measured the growth of cells of each mutant strain against that of wild-type P. aeruginosa PA14 encapsulated in LB nutrient media and in 1% agarose gels (infused with LB nutrient media). We first normalized the absorbance (λ = 595 nm) values of wild-type PA14 cells grown in liquid LB and those grown in 1% agarose and used these data to determine a percent growth value for each mutant compared to the wild-type strain. We determined a relative stiffness score—which we refer to here as a GRABS score (17)—for all mutant strains against wild-type PA14 using the following equation:

$$\text{GRABS score}=100\;\times \left(\frac{{\text{OD}}_{\text{mutant, agarose}}}{{\text{OD}}_{\text{wild type, agarose}}}-\frac{{\text{OD}}_{\text{mutant, LB}}}{{\text{OD}}_{\text{wild type, LB}}}\right)$$

A positive GRABS score for a mutant indicates that if wild-type and mutant cells grow at the same rate in liquid LB, cells of the mutant grow faster in 1% agarose than wild-type cells. On the basis of our past experiments, a positive GRABS score for a mutant indicates cells with increased stiffness. A negative GRABS score for a mutant indicates cells with reduced stiffness (compared to wild-type cells) and that mutant cells grow more slowly in 1% agarose than wild-type cells (again, when the growth rates of wild-type and mutant in liquid LB are similar).

For our screen in LB medium supplemented with d-Ala, we normalized all mutant data to an average of several wild-type control growth curves in liquid and agarose infused with plain LB. We used multiple wild-type controls on each plate to normalize the data.

### Gene ontology analysis.

We performed phylogenetic classification of the P. aeruginosa stiffness regulators by grouping them into various COGs categories. We got COGs information from the Pseudomonas Genome Database (http://www.pseudomonas.com) (45). We counted the number of genes in individual COGs category for the gene ontology analysis whose results are presented in Fig. 3C.

### Construction of the *dadA* and *dadAX* mutants.

The Δ(*dadA*) and Δ(*dadAX*) in-frame deletion strains were constructed as described previously (72) using Lambda Red recombination in which pAS03 was used for the FLP recombination target (FRT)-aacC1-FRT template, pUCP18-RedS (73) was used to express the recombineering machinery in PA14 cells, and p pFLP2 (74) was used to flip out the antibiotic resistance gene. For the *Δ*(*dadA*) strain, the region approximately 500 bp upstream of the sequence encoding DadA through the start codon of *dadA* (referred to as the *dadA*′ region) was amplified using PCR and primers 5′ GGTTACCTTGGCGTGGTCAG 3′ (dadA-u1) and 5′ CATTGTCGCCTCCCACGTCG 3′. The sequence encoding the final 18 amino acids, the translational stop codon of *dadA*, and approximately 500 bp downstream of the stop codon (referred to as the ′*dadA* region) was amplified using primers 5′ CATCCAGCGCCAGCACACTA 3′ and 5′ TCCGCTTTAATCACCGCGAG 3′ (dadA-l3). The FRT-aacC1-FRT region in pAS03 was amplified through PCR using primers 5′ AAACGTCCGCGGTTCCTCCGCGACGTGGGAGGCGACAATGattccggggatccgtcgacc 3′ (dadA-u2) and 5′ GATCTGGCTCATACGCTCGTTAGTGTGCTGGCGCTGGATGtgtaggctggagctgcttcg 3′, where the bases indicated with lowercase letters are identical to those in priming sites P4 and P1 of pAS03, respectively, and the uppercase letters are identical to bases that overlap the *dadA′* region or ′*dadA* region, respectively. The dadA′-FRT-aacC1-FRT-′dadA product was assembled through isothermal assembly, amplified using the dadA-u1 and dadA-l3 primers, transformed into PA14 expressing the plasmid pUCP18-RedS, and selected for gentamicin resistance, yielding IPF01. The antibiotic resistance marker was flipped out by electroporating IPF01 with p pFLP2, plating on LB plates containing 200 µg/ml carbenicillin, and restreaking on LB plates containing 5% sucrose to counterselect against p pFLP2, yielding markerless *Δ*(*dadA*) strain IPF01.1. For the Δ(*dadAX*) strain, the region containing the sequence encoding the final 18 amino acids of DadA, the translational stop codon, and approximately 600 bp downstream of the stop codon (referred to as the ′*dadX* region) was amplified using primers 5′ CGCGTCTATTCCGGGGCTTG 3′ and 5′ ACATAGGTCTCGCTGCCAAC 3′ (dadX-l3). The FRT-aacC1-FRT region in pAS03 was amplified for this construct through PCR using the dadA-u2 primer and 5′ AGAATTCGGAAAGTTTTTCTCAAGCCCCGGAATAGACGCGtgtaggctggagctgcttcg 3′, where the uppercase bases are identical to the ′*dadX* region and the lowercase bases are identical to the P1 priming site. The dadA′-FRT-aacC1-FRT-′dadX product was assembled through isothermal assembly, amplified using the dadA-u1 and dadX-l3 primers, and transformed into PA14 as described above, yielding IPF02. The antibiotic resistance marker was flipped out using p pFLP2, and the plasmid was cured to produce markerless strain IPF02.1.

### Complementation and deletion assays for *dadA* mutants.

We acquired a *dadA*::Tn mutant strain complemented with pDadAX plasmid (48). A pDadAX plasmid was constructed using pUCP18 vector, which enables constitutive expression of *dadA* from the *lac* operon promoter. We also used PA14 wild-type/pUCP18 and *dadA*::Tn/pUCP18 strains as empty vector controls for GRABS measurements. Briefly, we streaked strains complemented with different plasmids onto LB agar plates with appropriate antibiotics to obtain individual colonies. Individual colonies were inoculated into 2 ml of LB containing appropriate antibiotics and grown overnight at 37°C. GRABS 96-well plates were prepared as described above. For complementation assays, to avoid influencing the growth of cells, we did not add antibiotics.

### Microfluidics-based stiffness measurements.

P. aeruginosa PA14 wild-type, *dadA*::Tn, and *dadA*::Tn/pDadAX strains were grown overnight in 2 ml of LB containing appropriate antibiotics. We diluted overnight cultures 1:100 in fresh LB without any antibiotics and used 5 µg/ml aztreonam to filament both P. aeruginosa PA14 wild-type cells and *dadA*::Tn cells for 2 h. We also confirmed that the wild-type and *dadA*::Tn cells showed similar levels of sensitivity to aztreonam and that the MIC of aztreonam was 12.5 µg/ml (see Fig. S7 in the supplemental material). The absorbance (λ = 600 nm) of filamented cells was normalized to 1.0 before loading into individual channels of the microfluidic flow device was performed to monitor deflection under conditions of fluid flow. We determined the bending rigidity and Young’s modulus of cells by deflecting filamented wild-type and mutant strains under conditions of fluid flow in a microfluidic flow device as described previously (54). Images were collected using a Zeiss Axiovert 100 microscope equipped with a 63× oil objective equipped with an Andor iXon 3 electron-multiplying charge-coupled device (EMCCD). Deflection of the cells was determined using a custom Igor Pro (WaveMetrics Inc.) image analysis algorithm, and bending rigidity values were obtained via curve fitting.

10.1128/mBio.01340-18.7 FIG S7  Wild-type PA14, *dadA*::Tn mutant, and Δ*dadA* strains have nearly identical sensitivities to aztreonam. Download FIG S7, PDF file, 0.4 MB. Copyright © 2018 Trivedi et al. 2018 Trivedi et al. This content is distributed under the terms of the Creative Commons Attribution 4.0 International license.

To measure the diameter of mutant cells compared to wild-type cells after filamentation, we collected static images with a 100× oil objective on a Nikon Eclipse Ti inverted microscope equipped with a CoolSNAP HQ2 camera and determined cell widths using ImageJ. We determined that the cell width of wild-type cells was 1 µm, which was similar to the width quantified for the *dadA*::Tn mutant. These measurements were used to calculate the Young’s modulus from the flexural rigidity, for which the moment of inertia (*I*) of a cross-section is dependent on cell radius (*r*) and thickness of the cell wall (*h*) according to the equation *I =* π*r*^3^*h*.

### Determining the MIC of DCS.

We used the microdilution protocol to determine the MIC in accordance with Clinical and Laboratory Standards Institute guidelines (75). Briefly, we added 100 mM DCS to the first well of a 96-well microplate and diluted DCS 2-fold across adjacent wells (wells 1 to 11); well 12 was a no-drug control. We determined the MIC after 16 h of cell growth at 30°C with shaking by identifying by visual inspection the lowest concentration of DCS that inhibited cell growth. For all the MIC experiments, we grew cells in LB media with no antibiotic selection marker present, and the MIC was determined from three replicate plates.

### Quantitative PCR.

A Zymo Research Direct-zol RNA Miniprep kit was used to extract total RNA. Genomic DNA was removed using ArcticZymes HL-dsDNase, and RNA was reverse transcribed using an Applied Biosystems High Capacity RNA-to-cDNA kit. Newly synthesized cDNA was treated with New England Biolabs RNase H to digest RNA hybridized to cDNA. Thermo Fisher PowerUp SYBR green master mix was used for quantitative PCR on an Applied Biosystems 7500 Fast real-time PCR system, following the manufacturer’s instructions for a standard cycling protocol. Primers for *rpsl* were used as endogenous controls.

### Purification of sacculi and ultraperformance liquid chromatography (UPLC) of peptidoglycan composition.

Overnight cultures of P. aeruginosa PA14 wild-type cells and *dadA* transposon mutant cells corresponding to an OD of 100 (λ = 600) were harvested by centrifugation at 5,000 × *g* for 10 min at 25°C and resuspended in 3 ml of LB. Cells were lysed with a tip sonicator (Qsonica) for 15 s and then 10 s at a power setting of 75. Boiling sodium dodecyl sulfate (SDS) was added to cell suspensions, and the reaction mixtures were stirred at 500 rpm for 3 h to mix the cell suspensions and SDS. Insoluble material was collected by several rounds of ultracentrifugation at 400,000 × *g* for 20 min at 25°C. Samples were prepared for UPLC as previously described (76) and injected into a Waters H class UPLC system equipped with a ethylene bridged hybrid (BEH) C_18_ column (Waters, MA) (1.7-µm pore size), using previously described elution conditions (70). Peaks were quantified and identified per the table, and cross-linking density and strand length data were calculated (77, 78).

10.1128/mBio.01340-18.8 FIG S8  GRABS score for P. aeruginosa wild-type cells, *dadA*::Tn cells, and *ΔdadA and ΔdadAX* strains. Download FIG S8, PDF file, 0.2 MB. Copyright © 2018 Trivedi et al. 2018 Trivedi et al. This content is distributed under the terms of the Creative Commons Attribution 4.0 International license.
