# Supplementary figures and images for: Mechanical Genomic Studies Reveal the Role of d-Alanine Metabolism in Pseudomonas aeruginosa Cell Stiffness
Source: mBio. 2018 Sep 11;9(5):e01340-18. doi: 10.1128/mBio.01340-18 (PMC6134093; doi:10.1128/mBio.01340-18)

**Fig. S1.** Subcellular localization of 36 genes with largest negative GRABS score

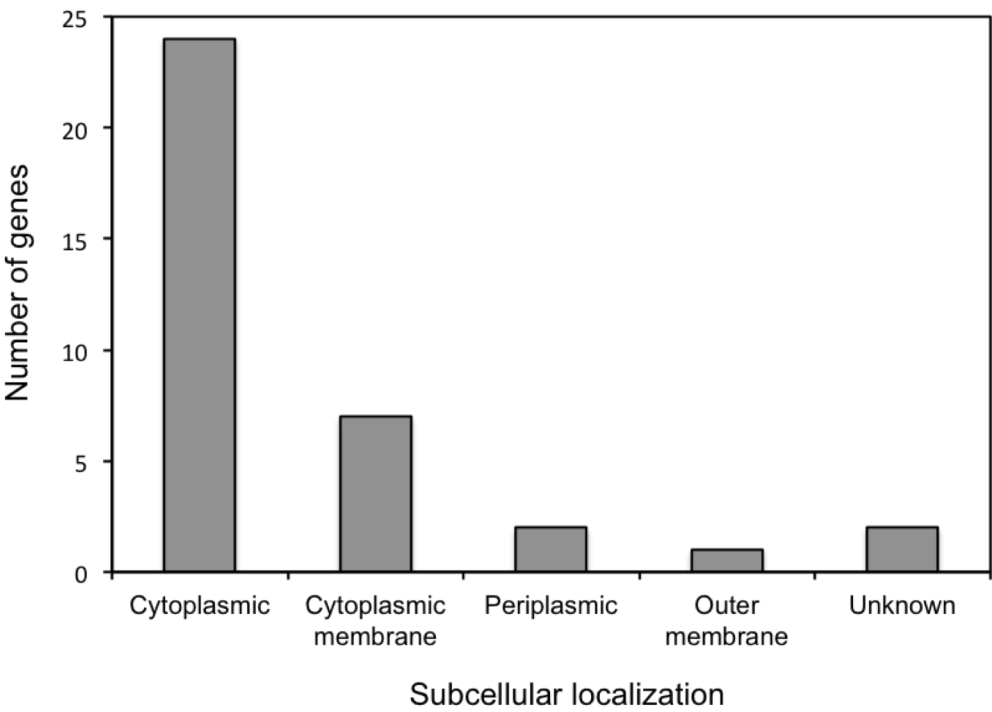

Supplement: FIG S1 [file mbo004184041sf1.pdf]

**Fig. S2.** DadX does not contribute to the stiffness

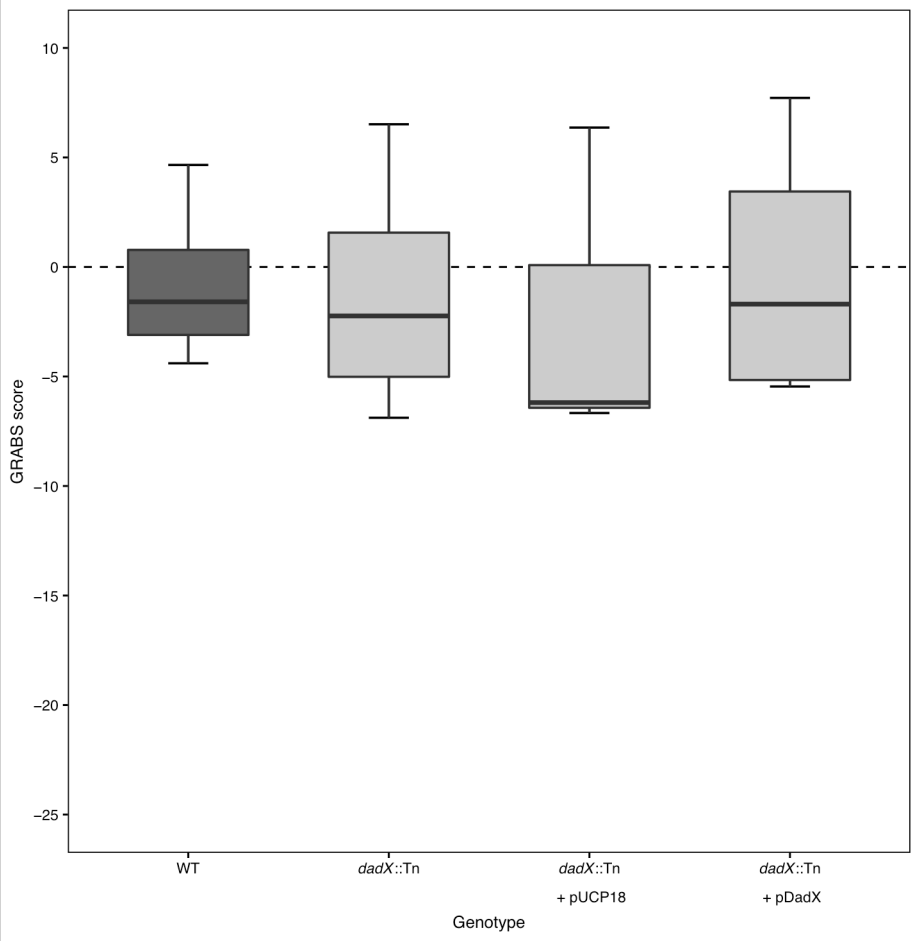

Supplement: FIG S2 [file mbo004184041sf2.pdf]

**Fig. S3.** In the presence of L-Ala, *dadA*::Tn and wild type cells have similar sensitivity against DCS

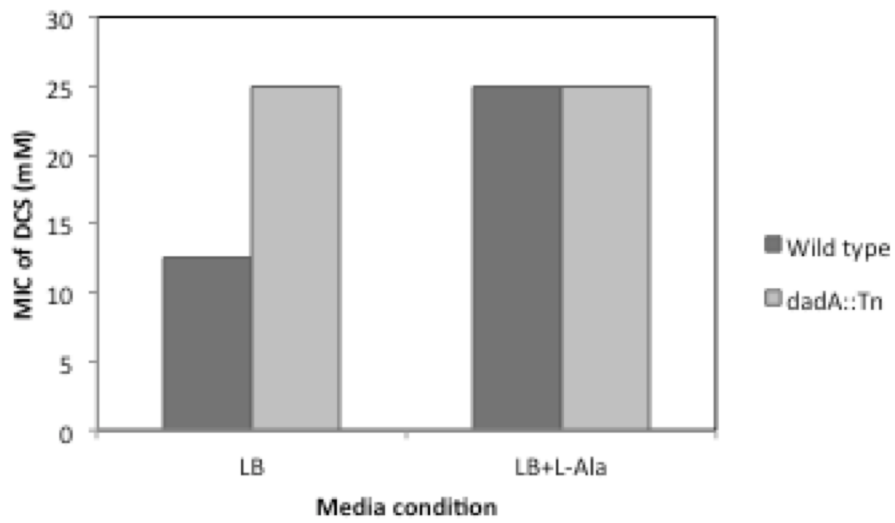

Supplement: FIG S3 [file mbo004184041sf3.pdf]

**Fig. S4.** GRABS score of *dadA*::Tn mutant does not change when grown in the presence of L-Ala.

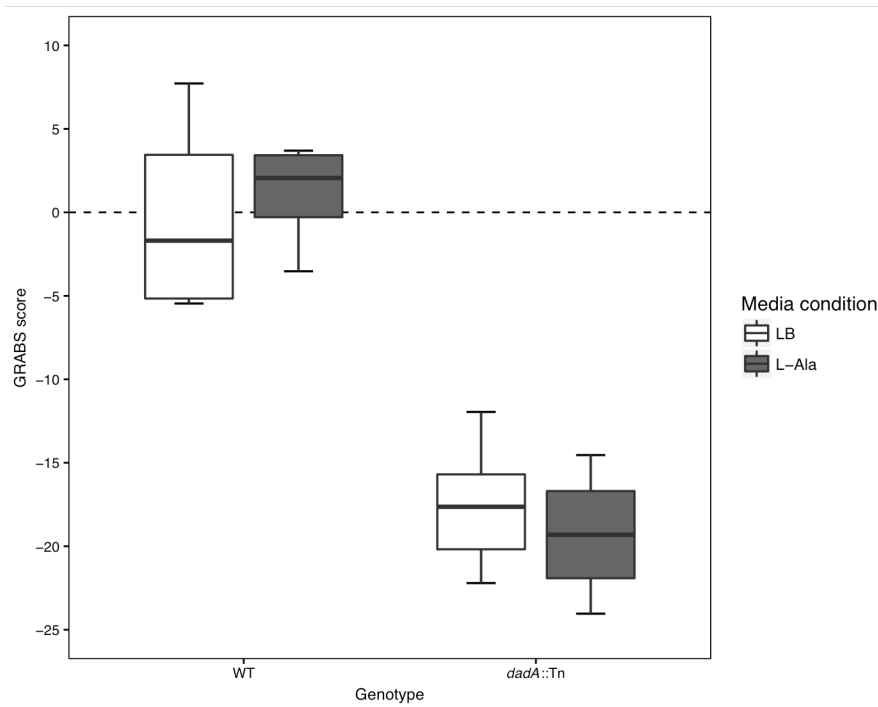

Supplement: FIG S4 [file mbo004184041sf4.pdf]
