# Supplementary material for: Mechanical Genomic Studies Reveal the Role of d-Alanine Metabolism in Pseudomonas aeruginosa Cell Stiffness
Source: mBio. 2018 Sep 11;9(5):e01340-18. doi: 10.1128/mBio.01340-18 (PMC6134093; doi:10.1128/mBio.01340-18)

Fig. S5. Wild type *P. aeruginosa* PA14 cells have higher dadA transcription when LB media is supplemented with 25mM D-Ala

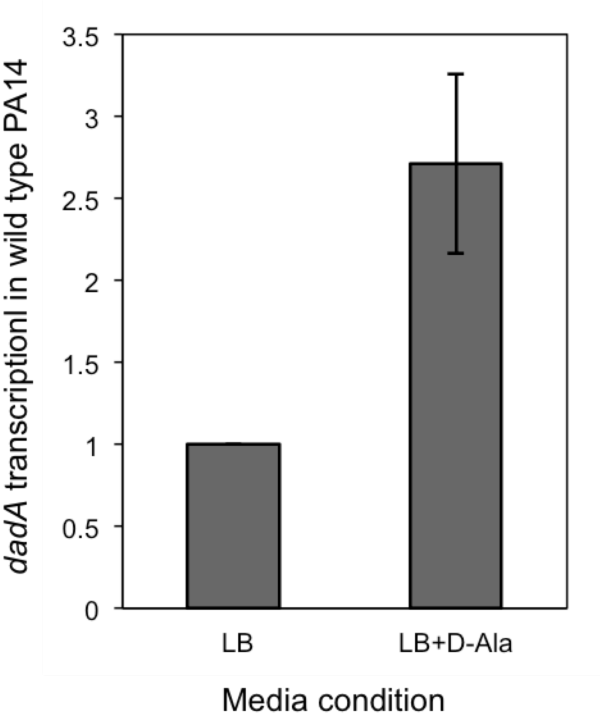

Supplement: FIG S5 [file mbo004184041sf5.pdf]
