# Supplementary material for: Mechanical Genomic Studies Reveal the Role of d-Alanine Metabolism in Pseudomonas aeruginosa Cell Stiffness
Source: mBio. 2018 Sep 11;9(5):e01340-18. doi: 10.1128/mBio.01340-18 (PMC6134093; doi:10.1128/mBio.01340-18)

Fig. S6. *dadA*::Tn has lower Young's modulus when the growth media is supplemented with D-Ala or D-Ala-D-Ala

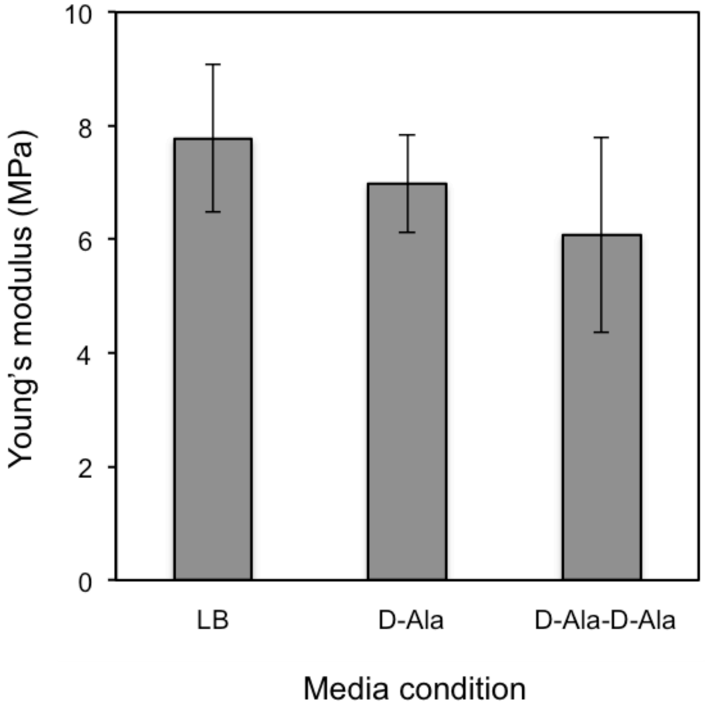

Supplement: FIG S6 [file mbo004184041sf6.pdf]
