# Supplementary material for: Mechanical Genomic Studies Reveal the Role of d-Alanine Metabolism in Pseudomonas aeruginosa Cell Stiffness
Source: mBio. 2018 Sep 11;9(5):e01340-18. doi: 10.1128/mBio.01340-18 (PMC6134093; doi:10.1128/mBio.01340-18)

Fig. S7. Wild type PA14, dadA::Tn mutant, and ΔdadA strains have nearly identical sensitivities to aztreonam

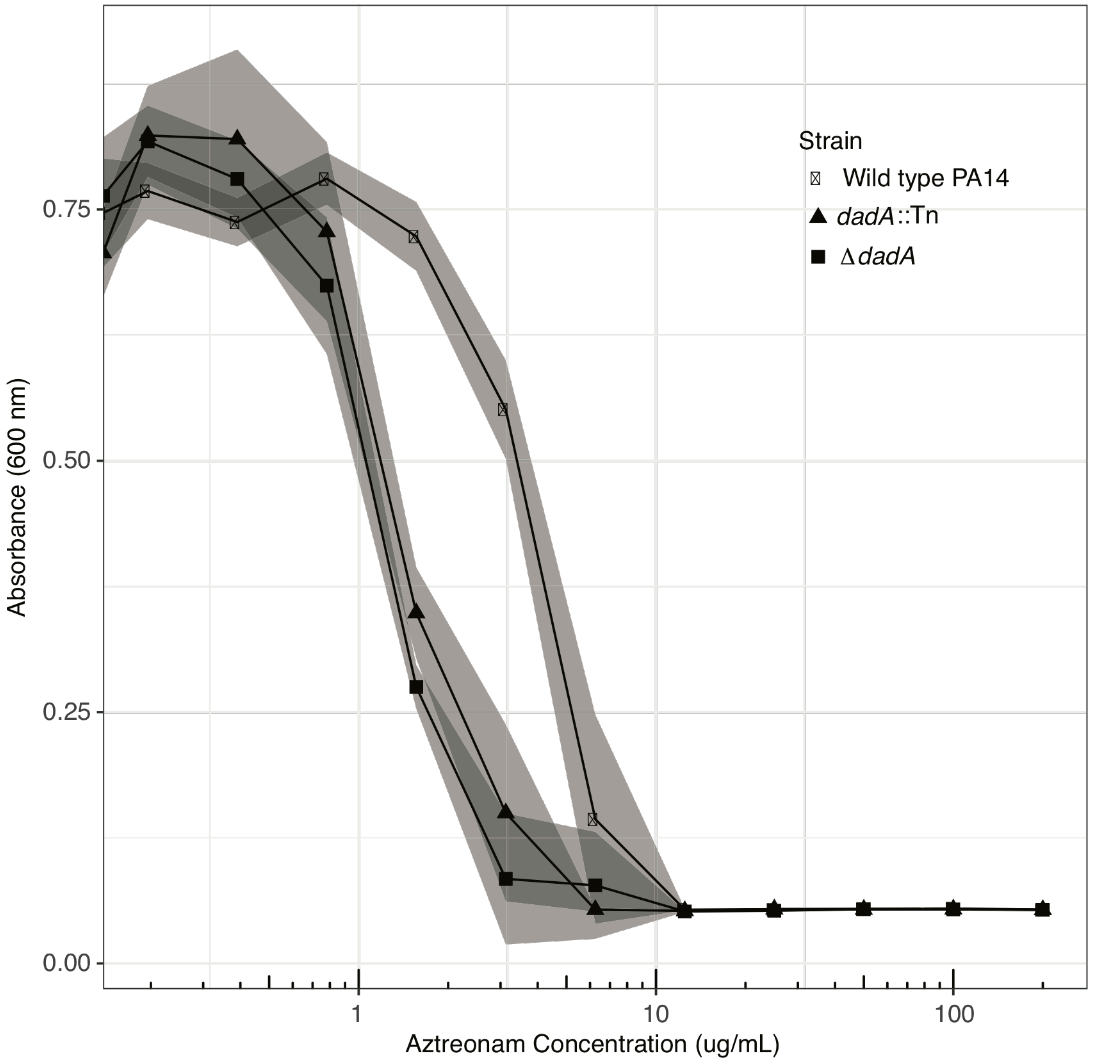

Supplement: FIG S7 [file mbo004184041sf7.pdf]
