# Supplementary material for: Mechanical Genomic Studies Reveal the Role of d-Alanine Metabolism in Pseudomonas aeruginosa Cell Stiffness
Source: mBio. 2018 Sep 11;9(5):e01340-18. doi: 10.1128/mBio.01340-18 (PMC6134093; doi:10.1128/mBio.01340-18)

**Fig. S8.** GRABS score for *P. aeruginosa* wild type cells, *dadA*::Tn cells, *ΔdadA, and ΔdadAX* strains

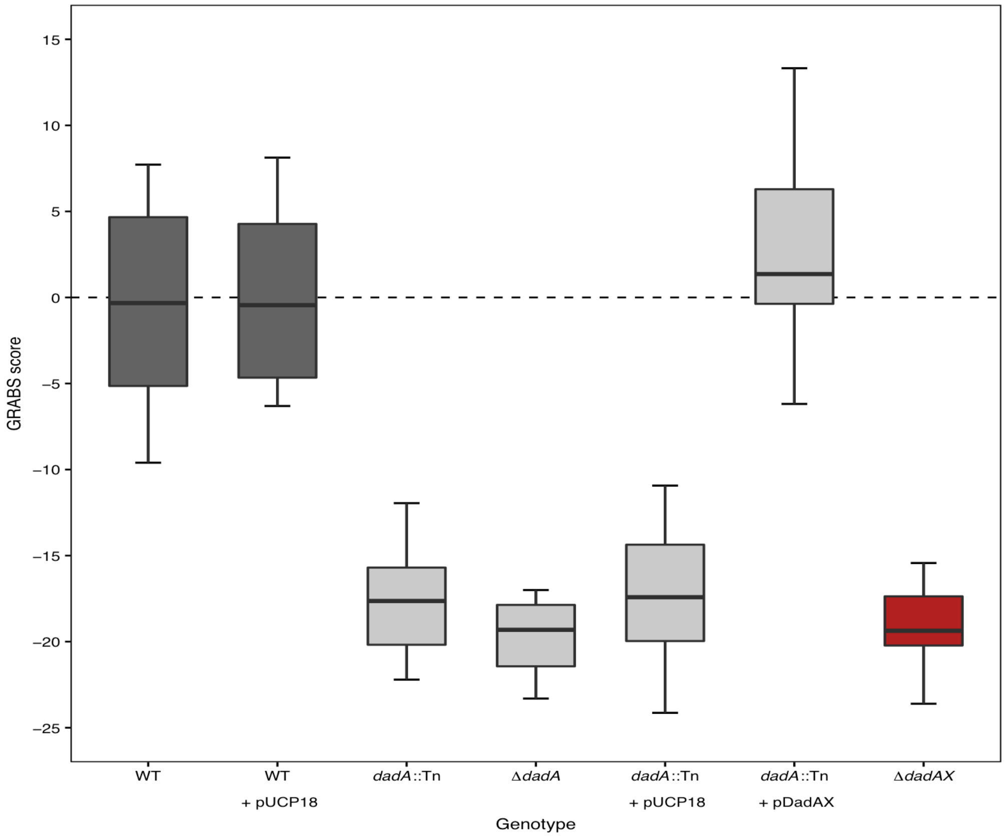

Supplement: FIG S8 [file mbo004184041sf8.pdf]
